# An unusual association of ulnar hemimelia with mesoaxial synostotic syndactyly

**DOI:** 10.1259/bjrcr.20190073

**Published:** 2020-02-12

**Authors:** Meltem Özdemir, Rasime Pelin Kavak, Hatice Kaplanoğlu

**Affiliations:** 1 University of Health Sciences, Dışkapı Yıldırım Beyazıt Training and Research Hospital, Department of Radiology, Ankara, Turkey

## Abstract

Ulnar hemimelia, also referred to as post-axial longitudinal deficiency of the upper limb, is a very rare skeletal anomaly characterized by the partial or complete absence of the ulna. The majority of the reported cases are sporadic and more common in males. The disorder is mostly unilateral, right-sided and incomplete. A slight shortening of the forearm, radial bowing and ulnar-sided hand drift are the anomalies which often accompany ulnar hemimelia. Ulnar hemimelia may also be seen in association with complex wrist and hand anomalies. The absence of post-axial metacarpal and digital bones are frequent findings in patients with this rare disorder. Cases with additional digital abnormalities such as post-axial syndactyly and camptodactyly are also present in the literature. However, a case of ulnar hemimelia in association with mesoaxial synostotic syndactyly has never been reported to date.

## Case presentation

A 20-year-old male with left upper limb disability admitted to Dışkapı Yıldıım Beyazıt Training and Research Hospital for mandatory health screening before military service. He had no remarkable symptoms or signs indicative of else pathology other than the skeletal disorder involving his left upper limb. He is the second of three sons born to non-consanguineous healthy parents. His brothers do not have any skeletal or other systemic disorders. There is no family history of any kind of congenital skeletal abnormalities in the extended family. His mother was at the age of 27 when she gave birth to our patient. There is no history of any drug, smoke, alcohol or radiation exposure during pregnancy. Our patient was born uneventfully at full-term through normal vaginal delivery. No other significant health problem is present in the history of his childhood. He stated that he had never received surgical or non-surgical treatment for the deformities involving his left upper extremity.

**Figure 1.**
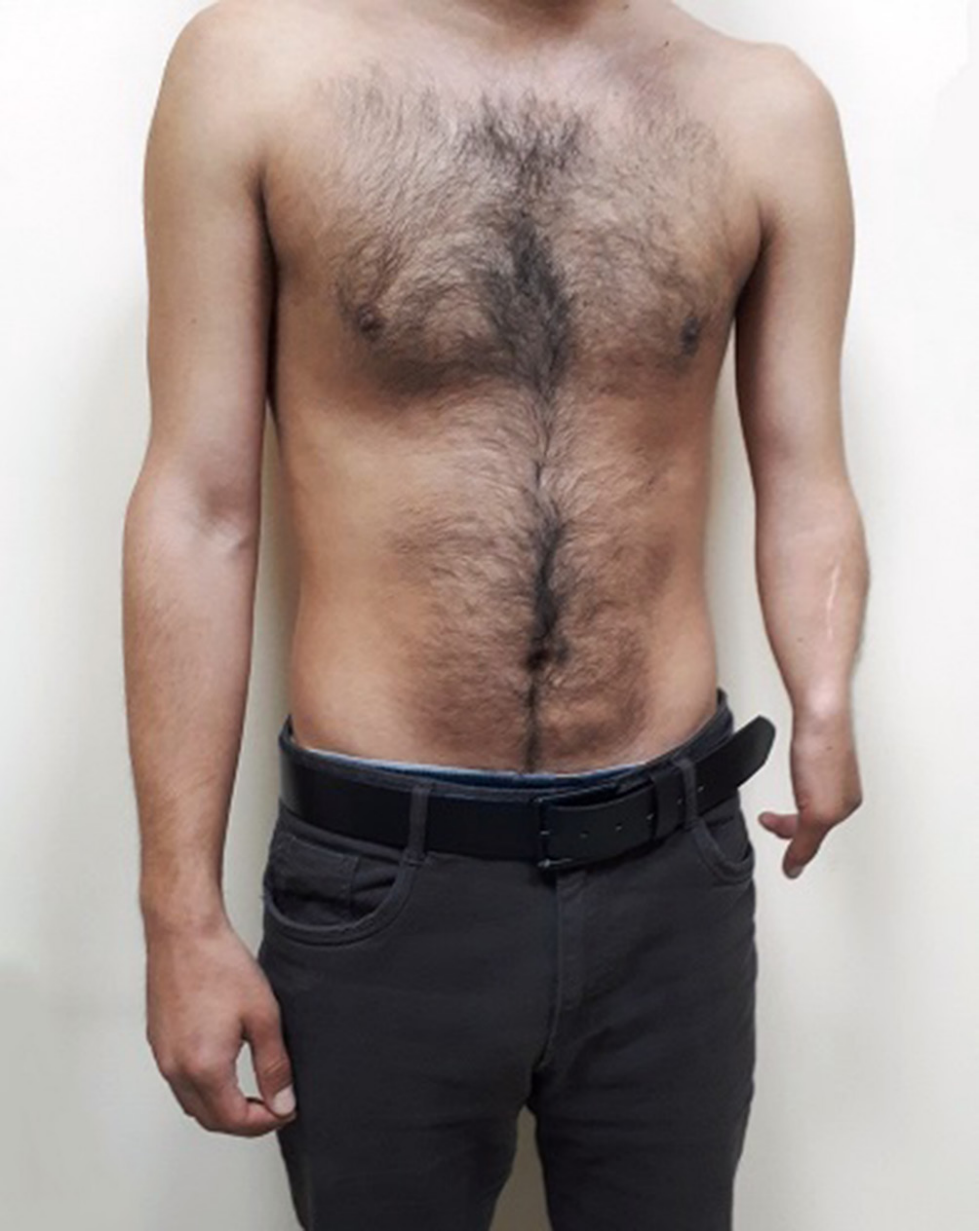
Clinical photograph of the patient with left ulnar hemimelia.

**Figure 2.**
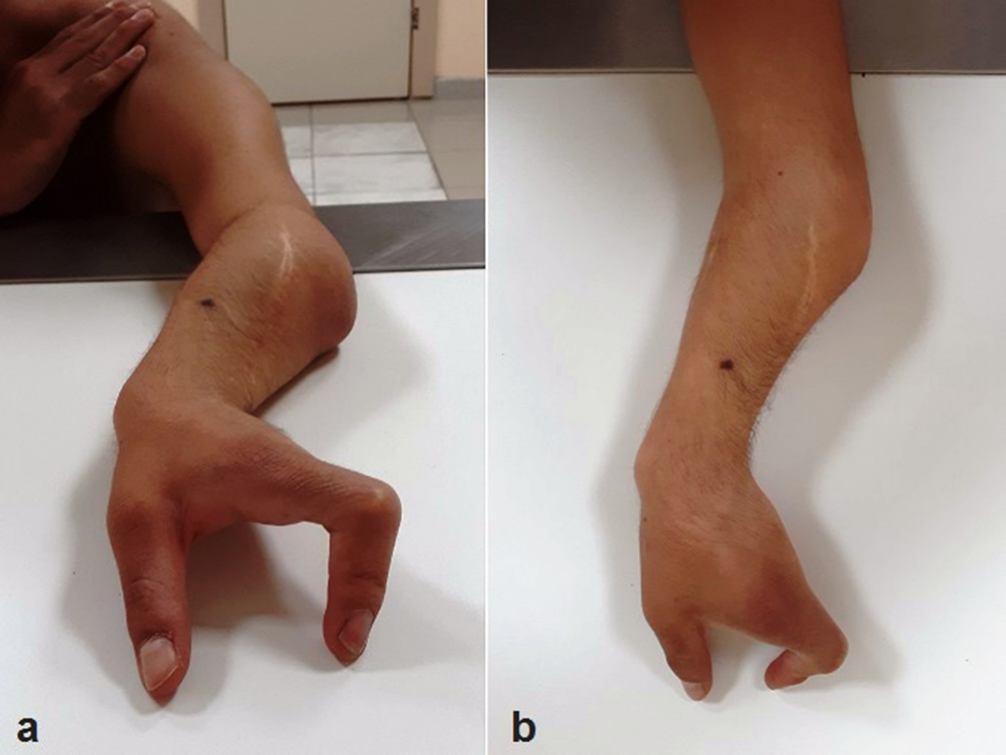
Clinical photographs of the left upper limp of the patient with ulnar hemimelia showing a slight flexion of the elbow joint, mild radial bowing, ulnar-sided hand drift and oligodactyly. Note that the skin and nails of the existing fingers are normal.

On physical examination, his left upper limb was short and forearm was slightly curved (Figure 1). There was a slight fixed-flexion of the elbow joint. Left hand oligodactyly and a mild ulnar-sided drift of the left hand were also noted (Figure 2). Using his left hand, he was unable to perform most of the manual activities but was capable of grip.

## Imaging findings

**Figure 3.**
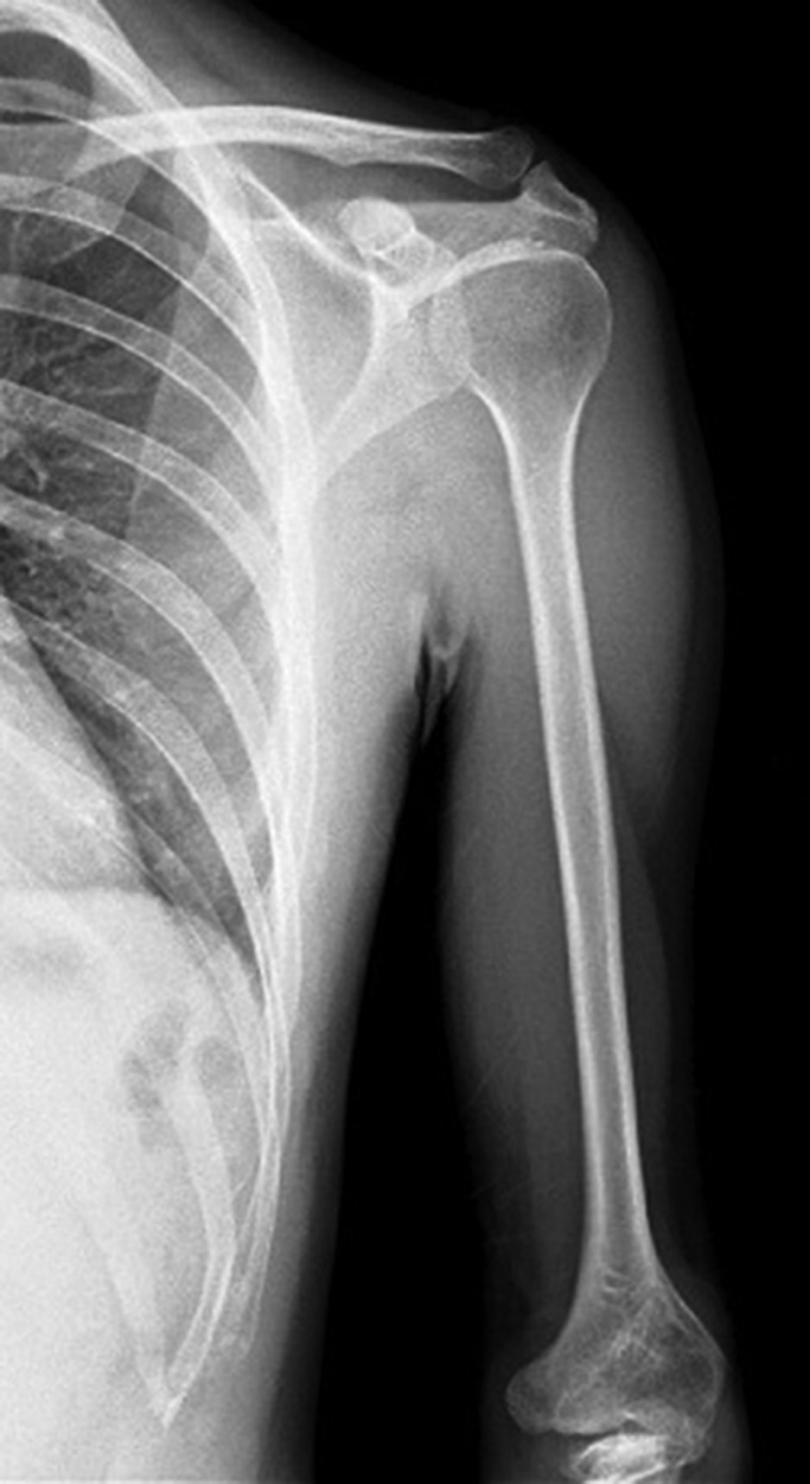
Anteroposterior radiograph of the left arm showing normal shoulder joint and humerus.

**Figure 4.**
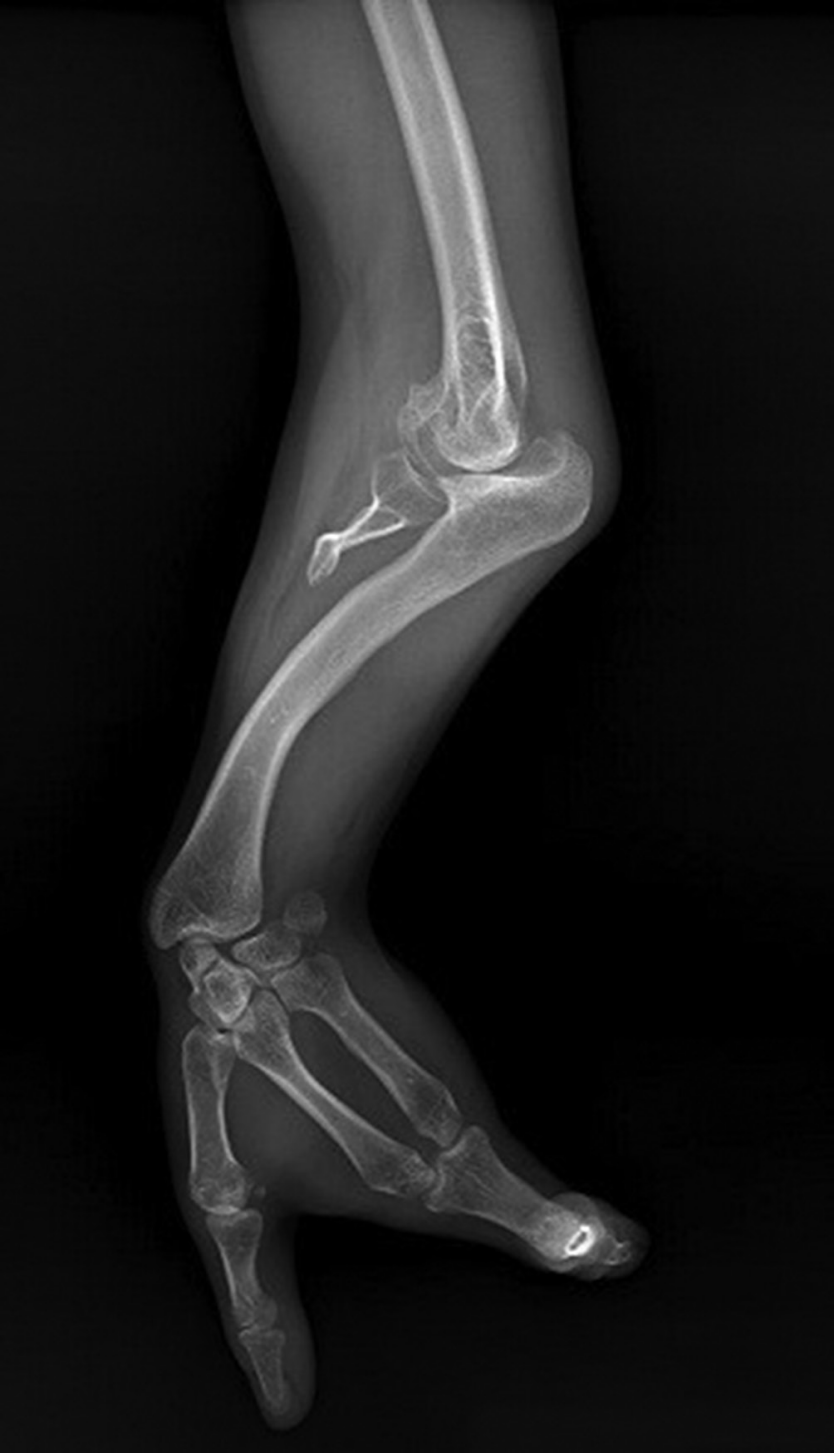
Anteroposterior radiograph of the left forearm demonstrating the absence of the ulnar bone distal to its rudimentary proximal segment. Radial bowing with the curve convex to the medial side is also evident.

**Figure 5.**
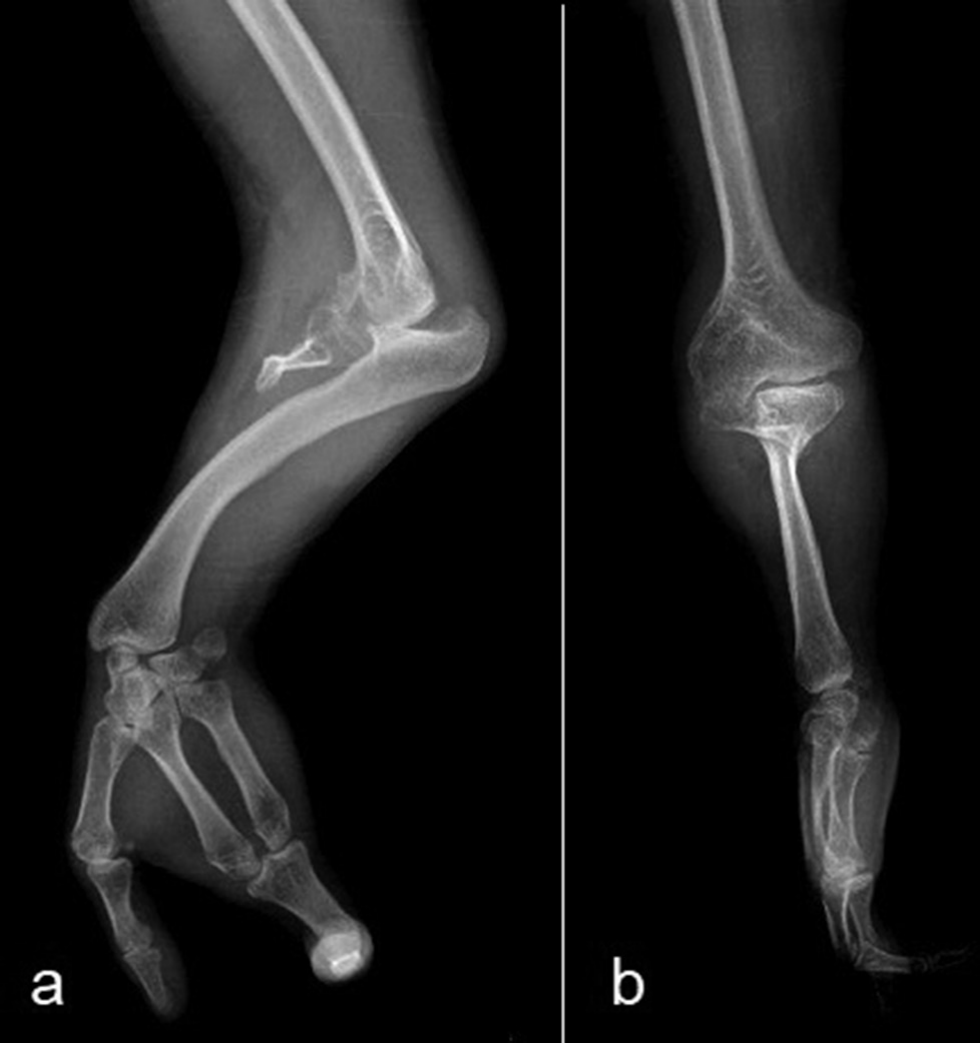
Biplanar left elbow radiographs demonstrating fixed flexion of about 45° of the elbow accompanied by degenerative changes in the humeroradial joint.

**Figure 6.**
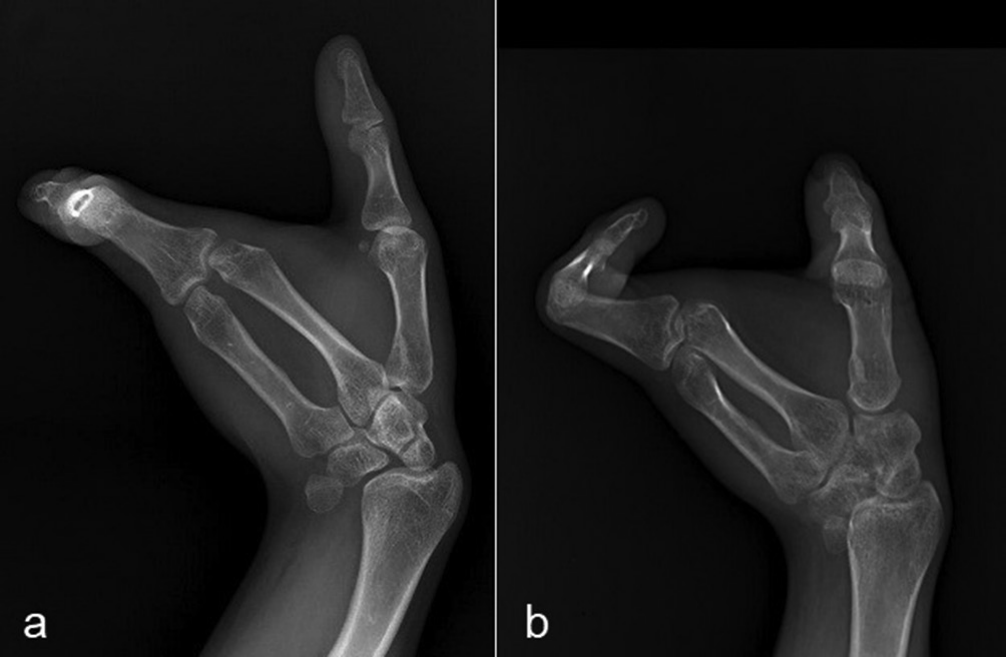
Biplanar left wrist and hand radiographs showing the absence of ulnar-sided carpal bones, ulnar-sided hand drift, postaxial oligodactyly and mesoaxial synostotic syndactyly.

Anteroposterior radiograph of the left arm showed that the shoulder joint and humerus were normal (Figure 3). Anteroposterior radiograph of the left forearm demonstrated that the proximal ulnar segment participating in the elbow joint structure was small but present, while the ulna distal to this point was absent. Radial bowing with the curve convex to the medial side was also noted (Figure 4). Dedicated left elbow radiographs revealed fixed flexion of about 45° of the elbow accompanied by degenerative changes in the humeroradial joint (Figure 5). The absence of ulnar-sided carpal bones and ulnar-sided hand drift were recorded on the dedicated wrist and hand radiographs (Figure 6). The 4*–*5th metacarpals as well as fingers were absent (post-axial oligodactyly). There was symmetric triangular-shaped synostosis of the proximal phalanges of the 2*–*3rd fingers while the respective metacarpals were normal (mesoaxial synostotic syndactyly). There were two metacarpophalangeal but only one proximal interphalangeal joint in this synostotic finger. Fixed flexion of the proximal interphalangeal (camptodactyly) and fixed extension of the distal interphalangeal joints of the synostotic finger were evident on lateral hand radiograph. The thumb was normal.

Based on the characteristic radiographic findings, the patient was diagnosed as having ulnar hemimelia with postaxial oligodactyly and mesoaxial synostotic syndactyly.

## Treatment

A regular and continuous physiotherapy plan was arranged in order to optimize the functionality of the elbow joint and prevent the development of disuse atrophy. However, no surgery was planned.

## Discussion

Hemimelia is the name given to the congenital skeletal anomaly that presents as the absence or underdevelopment of one side of the distal half of a limb. Ulnar hemimelia, also referred to as post-axial longitudinal deficiency of the upper limb, occurs at a rate of about 1 in 150,000 live births. It is a very rare type of hemimelia in which the ulna is completely or partially absent.^1^ Different from the radial hemimelia, ulnar hemimelia is mostly non-syndromic and the majority of the reported cases are sporadic. It may also appear as a component of skeletal dysplasias. In rare cases, ulnar hemimelia may present with some syndromes such as Poland, Goltz-Gorlin, Cornelia De Lange, or Femur Fibula Ulna syndromes. The disorder is more common in males compared to females. In approximately 70% of the cases, ulnar hemimelia is unilateral, mostly right-sided and incomplete.^2^

Ulnar hemimelia presents as the hypoplasia of the forearm accompanied by complex elbow and carpus abnormalities.^3^ Some shortening of the forearm, radial bowing and tendency of the hand to drift to the ulnar side of the wrist which usually accompany ulnar hemimelia were all present in the case we currently present. Other skeletal anomalies such as humeroradial synostosis, radial head dislocation and carpal coalition may also be seen in cases with ulnar hemimelia.^2–5^ While there were a humeroradial joint distance reduction and a fixed flexion of about 45° of the elbow joint in our patient, a characteristic humeroradial synostosis which typically causes fixed flexion or extension of the elbow joint was not present.

As an indication that post-axial wrist and hand halves are uniquely formed in response to an ulnarizing influence, the anatomy of the carpal, metacarpal and digital elements alters in almost all cases of ulnar hemimelia.^6^ It is a striking finding that there is an increased incidence of wrist and hand deficiency as one passes from the radial side to the ulnar side.^3^ So, the absence of post-axial carpal, metacarpal and digital bones are frequent findings in patients with ulnar hemimelia. In general, the degree of wrist and hand anomaly correlates with the degree of ulnar hypoplasia. The most common hand anomaly associated with the disorder is three-fingered hand, closely followed by monodigital hand.^3,4^ Cases with additional digital abnormalities such as postaxial syndactyly and camptodactyly are also present in the literature.^2^ Our patient presented a marked deficiency of the ulna. And parallel to the severity of the ulnar failure, postaxial carpal metacarpal and digital elements were all absent. Moreover, an unusual form of syndactyly involving the index and middle fingers was evident on the hand radiograph of the patient. A triangular shaped proximal phalanx was articulating with the second and third metacarpals and a single middle phalanx.

Recently, Weinrich et al reported a case of an isolated unilateral mesoaxial synostotic syndactyly and presented this unusual case as a novel nonsyndromic syndactyly of the hand.^7^ The phenotype of the digital anomaly of this case, which was almost the same as that of our patient, included a triangular proximal phalanx articulating with the second and third metacarpals and a single middle phalanx. Malik et al stated that this case was consistent with syndactyly type IX (OMIM #609432) and objected its presentation as a new syndactyly type.^8–10^ However, while syndactyly type IX is characterized by osseous synostosis of the third and fourth metacarpals, synostosis of the case of Weinrich et al was involving the second and third proximal phalanges. Therefore, this discussion did not result in a consensus.^11^ As syndactyly of our patient is not isolated but accompanies ulnar hemimelia, the present case is outside the limits of this discussion. What makes our case special is that it is the only reported case of mesoaxial synostotic syndactyly in association with ulnar hemimelia.

The clinical presentation and course of ulnar hemimelia is determined by the severity of the ulnar deficiency and the presence of other accompanying upper limb anomalies. While patients with an isolated mild ulnar deficiency may remain asymptomatic, cases with prominent ulnar deficiency accompanied by complex upper limb abnormalities present with a severe disability. Treatment of this disorder is difficult and should be highly personalized. Continous physiotherapy and training should be given from infancy in order to avoid disuse atrophy and to obtain maximum functioning of the limb.^2^ It is shown that the majority of surgeries for ulnar hemimelia are performed on the hand. Hand surgeries are planned for the proper function of the thumb and fingers. Syndactyly release, thumb rotation, modified pollicization procedures and rotational metacarpal osteotomies are surgical procedures performed to increase the ability to use the hand.^5,6^ In selected cases, various surgical approaches including Z-plasty, elbow disarticulation, and humeral derotation osteotomy are performed to improve the range of motion of the elbow joint.^2^ Our patient stated that he had never received physiotherapy or surgical treatment to date. In order to improve the elbow function and avoid disuse atrophy, a regular and continuous physiotherapy was planned. However, he was not suitable for any kind of surgical intervention and no surgery was planned.

## Consent for publication

Written informed consent was obtained from the patient for publication of this case report, including accompanying images

## Learning points

1. Ulnar hemimelia, also referred to as postaxial longitudinal deficiency of the upper limb, is a very rare skeletal anomaly characterized by the partial or complete absence of the ulna.
2. Some shortening of the forearm, radial bowing and an ulnar-sided hand drift usually accompany this rare disorder.
3. The absence of post-axial carpal, metacarpal and digital bones are frequent findings in patients with ulnar hemimelia.
4. Mesoaxial synostotic syndactyly, which is a very rare hand anomaly, may accompany ulnar hemimelia.
